# Molecular insights into the biochemical functions and signalling mechanisms of plant NLRs

**DOI:** 10.1111/mpp.13195

**Published:** 2022-03-30

**Authors:** Xiaoxiao Liu, Li Wan

**Affiliations:** ^1^ National Key Laboratory of Plant Molecular Genetics CAS Center for Excellence in Molecular Plant Sciences Institute of Plant Physiology and Ecology Chinese Academy of Sciences Shanghai China

**Keywords:** cell death, immune receptors, pathogen effector, plant immunity, signal transduction

## Abstract

Plant intracellular immune receptors known as NLR (nucleotide‐binding leucine‐rich repeat) proteins confer immunity and cause cell death. Plant NLR proteins that directly or indirectly recognize pathogen effector proteins to initiate immune signalling are regarded as sensor NLRs. Some NLR protein families function downstream of sensor NLRs to transduce immune signalling and are known as helper NLRs. Recent breakthrough studies on plant NLR protein structures and biochemical functions greatly advanced our understanding of NLR biology. Comprehensive and detailed knowledge on NLR biology requires future efforts to solve more NLR protein structures and investigate the signalling events between sensor and helper NLRs, and downstream of helper NLRs.

## Graphical abstract

Structural biology greatly advanced our understanding on the biochemical functions and signaling mechanisms of plant NLR proteins.

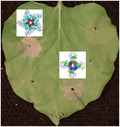

## 1 INTRODUCTION

Plants are constantly exposed to a wide variety of microbial pathogens, including bacteria, fungi, oomycetes, and viruses. To resist infections, plants have evolved an active two‐layered immune system. The first layer involves recognition of pathogen‐associated molecular patterns (PAMPs) by plant cell surface pattern recognition receptors (PRRs), known as PAMP‐triggered immunity (PTI). Successful pathogens secrete effectors into plant cells to suppress the PTI response and cause infections. Plants respond to pathogen effectors specifically with cytoplasmic NLR (nucleotide‐binding leucine‐rich repeat) protein receptors and activate effector‐triggered immunity (ETI), which constitutes the second layer of the plant immune system. The hallmark of ETI is localized plant cell death at infection sites, also known as the hypersensitive response, to restrict pathogen spread (Jones et al., 2016).

Knowledge of activation and signalling mechanisms of PRR and NLR receptors has greatly advanced (DeFalco & Zipfel, 2021; Saur et al., 2021). Increasing evidence suggests that the two layers of immunity are more intimately connected than previously thought (Ngou et al., 2021; Yuan et al., 2021). Research on the interdependency between PRR‐ and NLR‐meditated immunity is rapidly developing (Pruitt et al., 2021; Tian et al., 2021). NLR protein signalling mechanisms are also under intensive investigation after recent breakthrough studies revealing the unexpected biochemical functions of NLR proteins (Wang et al., 2020). Structural biology, specifically through advancements in cryoelectron microscope (cryo‐EM) techniques, has significantly contributed to our understanding of NLR protein biology. This review will focus on current knowledge and future perspectives on plant NLR protein activation and signalling.

## 2 NLR DOMAIN STRUCTURE AND SUBFAMILIES

**FIGURE 1:**
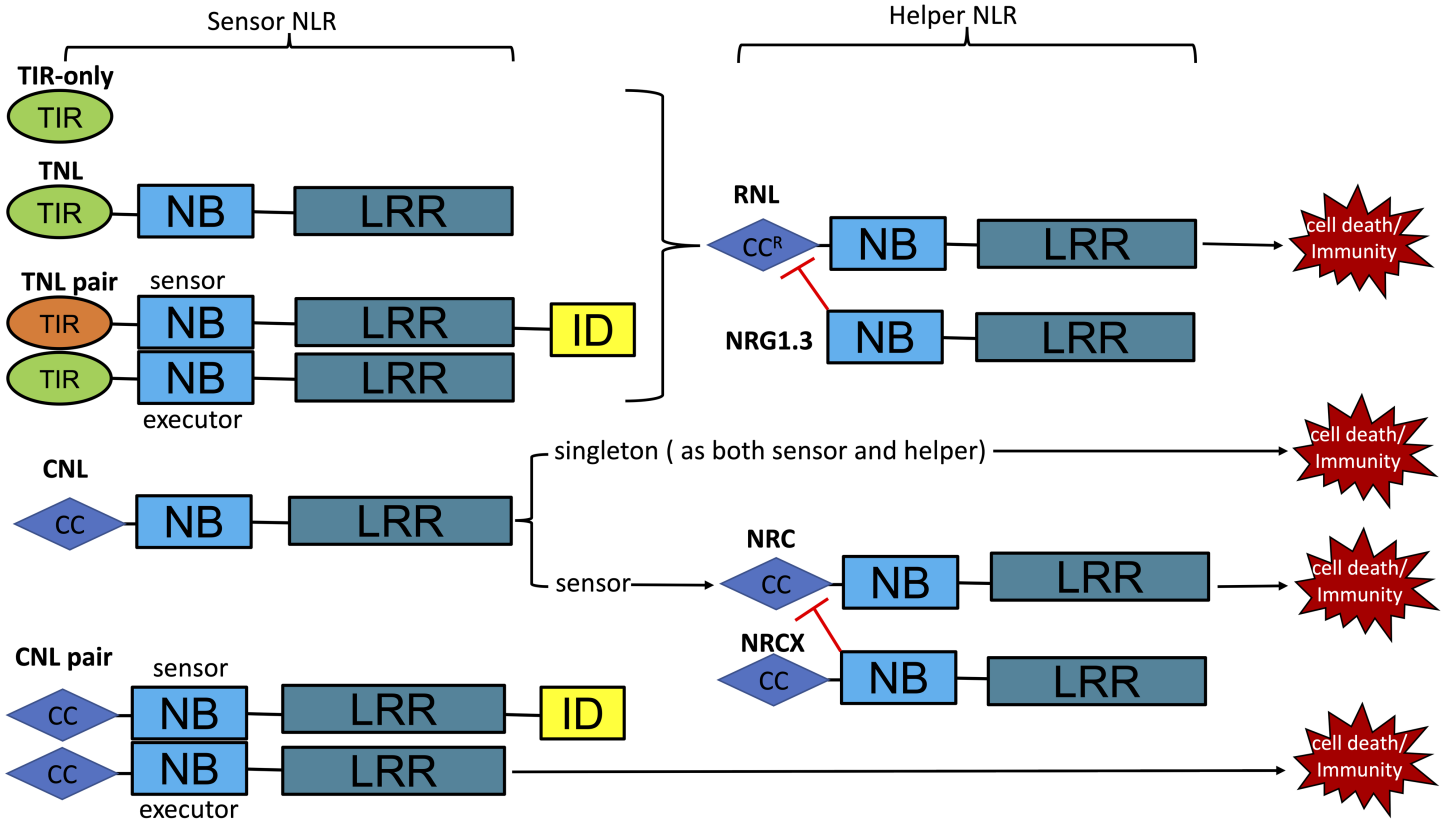
Schematic diagram depicting the domain structures of sensor NLRs and helper NLRs and their interdependence for function. TIR, toll/interleukin‐1 receptor; CC, coiled‐coil; CC^R^, (Resistance to Powdery Mildew 8)‐like CC; NB, nucleotide‐binding; LRR, leucine‐rich repeat; ID, integrated decoy

In addition to the central nucleotide‐binding (NB) domain and the C‐terminal leucine‐rich‐repeat (LRR) domain, plant NLR proteins have a variable N‐terminal domain that can be a Toll/interleukin‐1 receptor (TIR) domain or a coiled‐coil (CC) domain or an RPW8 (Resistance to Powdery Mildew 8)‐like CC (CC^R^) domain. NLR proteins with N‐terminal TIR, CC, or CC^R^ domains are known as TNLs, CNLs, or RNLs, respectively (Duxbury et al., 2021). TNLs and CNLs recognize pathogen effectors directly or indirectly to activate cell death and defence responses, thus are regarded as sensor NLRs (Jubic et al., 2019). TNLs or CNLs also function as pairs to perceive pathogen effectors and confer resistance. For instance, in *Arabidopsis* two TNLs, RPS4 and RRS1, located in a head‐to‐head orientation in the genome, cooperate to resist infection by at least three different pathogens (Narusaka et al., 2009). In rice, two CNLs, RGA4 (Resistance Gene Analogue 4) and RGA5 (Resistance Gene Analogue 5), function together to resist *Magnaporthe oryzae* carrying the effector Avr‐Pia or Avr1‐CO39 (Cesari et al., 2013). In the NLR pair system, one NLR possesses an additional domain called the integrated decoy (ID) domain for direct effector interaction functioning as a sensor, while the other NLR functions as an executor for immune signalling (Le Roux et al., 2015; Williams et al., 2014). Downstream of sensor NLRs or sensor/executor NLR pairs, higher plants also use helper NLRs to transduce signals. There are three described families of helper NLRs: NLR Required for Cell death (NRC), Activated Disease Resistance 1 (ADR1), and N Required Gene 1 (NRG1) (Jubic et al., 2019). In terms of domain structure, NRCs belong to the CNL class, while ADR1s and NRG1s belong to the RNL class. In solanaceous species such as *Nicotiana benthamiana*, NRCs are required for phylogenetically related CNLs to activate cell death and immune responses (Wu et al., 2017). In *N. benthamiana*, NRC2, NRC3, and NRC4 function downstream of different CNLs to positively regulate immunity, while NRCX negatively regulates NRC2 and NRC3‐mediated immunity (Adachi et al., 2021). In *Arabidopsis thaliana*, three ADR1s, NRG1.1 and NRG1.2 are full‐length RNLs (CC^R^‐NB‐LRR) functioning as positive regulators, while the truncated NRG1.3 (NB‐LRR) negatively regulates AtNRG1.1‐ and AtNRG1.2‐mediated immunity (Wu et al., 2021). In dicots, ADR1s and NRG1s are required downstream of TIR‐only receptors, TNLs and TNL pairs to activate defence responses, and ADR1s are also required for the full function of CNLs (Jubic et al., 2019). ADR1s, but not NRG1s, are also present in monocots with uncharacterized function. Some NLRs that function as both sensors of effectors and helpers directly activating cell death are regarded as NLR singletons (Adachi et al., 2019) (Figure 1).

## 3 NLR RECOGNITION OF EFFECTORS

**FIGURE 2:**
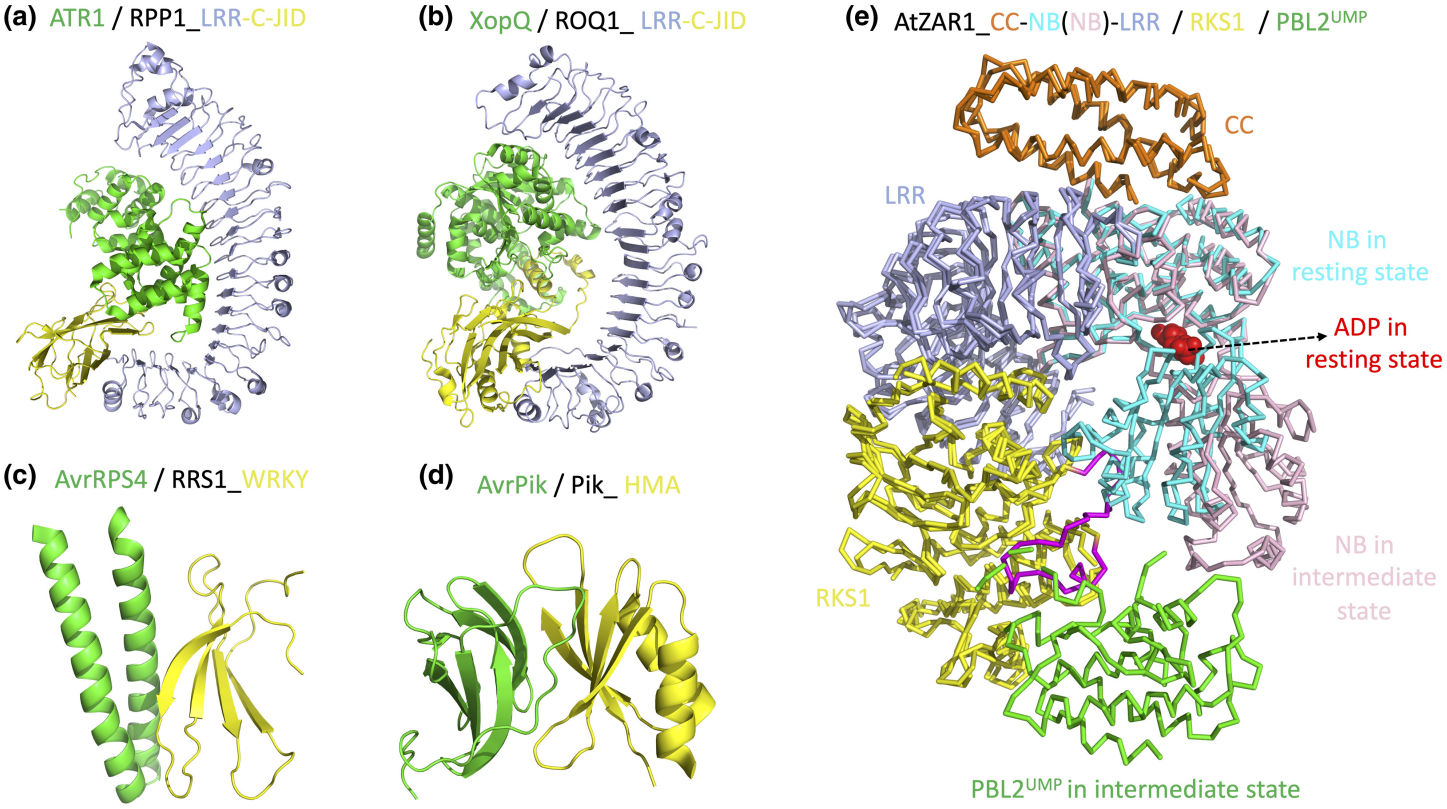
Current structure knowledge on NLR recognition of effectors. (a) Cryoelectron microscopy (cryo‐EM) structure of RPP1 LRR and C‐JID domains in a complex with effector ATR1 (PDB: 7CRB). (b) Cryo‐EM structure of ROQ1 LRR and C‐JID domains in a complex with effector XopQ (PDB: 7JLU). (c) Crystal structure of RRS1 WRKY domain in a complex with effector AvrRPS4 (PDB: 7P8K). (d) Crystal structure of Pik HMA domain in a complex with effector AvrPik (PDB: 5A6W). (e) Super‐imposition of the ADP‐bound ZAR1 structure in the resting state (6J5W) and the ADP‐free ZAR1 structure in the intermediate state (6J5V). Binding of effector‐produced PBL2^UMP^ causes RKS1 conformational changes (highlighted in purple) that clash with and push away the NB domain. The NB domain is shown in two colours (cyan and pink) to highlight the close‐to‐open switch that allows allosteric ADP release

Plant sensor NLRs recognize pathogen effectors via direct interaction or perceiving the modifications by effectors (Duxbury et al., 2021). Well‐known examples of direct NLR–effector recognition mostly come from TNLs including flax L567, *Arabidopsis* RPP1 (Recognition of *Peronospora parasitica* 1), and *N. benthamiana* ROQ1 (Recognition of XopQ 1). The structural bases of direct effector recognition, ATR1 by RPP1 and XopQ by ROQ1, have been reported (Ma et al., 2020; Martin et al., 2020). Interestingly, a previously undefined C‐terminal jelly roll/Ig‐like domain (C‐JID) was revealed in the post‐LRR domain regions of RPP1 and ROQ1. Both C‐JID and LRR domains are involved in effector recognition (Figure 2a,b). The structure‐conserved but sequence‐diversified C‐JID is present in many other TNLs from dicotyledonous plant species, but not in CNLs from diverse plant species (Ma et al., 2020).

In addition, well‐studied NLR pairs use the ID domain of sensor NLRs to physically associate with effectors for activation (Duxbury et al., 2021). In the TNL pair of RPS4 (Resistance to *Pseudomonas syringae* 4) and RRS1 (Resistance to *Ralstonia solanacearum* 1), the integrated WRKY domain of RRS1 detects effectors AvrRps4 from *Pseudomonas syringae* pv. *pisi* and PopP2 from *Ralstonia solanacearum* (Le Roux et al., 2015; Narusaka et al., 2009). Interestingly, crystal structures of RRS1 WRKY in complex with AvrRps4 or PopP2 revealed that the two effectors target the same region of RRS1 WRKY (Mukhi et al., 2021, Zhang et al., 2017b) (Figure 2c). In rice, two CNL pairs, RGA4/RGA5 and Pik‐1/Pik‐2, use the integrated heavy metal‐associated (HMA) domain in the sensors RGA5 and Pik‐1, respectively, to directly recognize cognate effectors. A few structures of the Pik‐1 HMA domain in complex with the effector Avr‐Pik highlighting the recognition specificities and key residues for activation have been reported (Bialas et al., 2021; De la Concepcion et al., 2018, 2019) (Figure 2d).

Well‐studied examples of the indirect effector‐recognition mode come from CNLs. In *Arabidopsis*, RPM1‐interacting protein 4 (RIN4) serves as a guardee physically bound to two CNLs, Resistance to *Pseudomonas syringae* 2 (RPS2) and Resistance to *Pseudomonas syringae* pv. *maculicola* 1 (RPM1). Effector‐induced cleavage or phosphorylation of RIN4 activates RPS2 and RPM1, respectively (Duxbury et al., 2021). Another well‐demonstrated example of indirect recognition is the *Arabidopsis* CNL ZAR1 (Hopz‐Activated Resistance 1) and the *Xanthomonas campestris* effector AvrAC. Two receptor‐like cytoplasmic kinases, RKS1 and PBL2, are involved in the recognition. RKS1 constitutively interacts with the LRR domain of ZAR1. AvrAC uridylates PBL2 and the uridylated PBL2 (PBL2^UMP^) is then recruited to the RKS1–ZAR1 complex to activate ZAR1 (Wang et al., 2015). The structures of the inactive RKS1‐ZAR1 complex (ADP bound) and the intermediate PBL2^UMP^‐RKS1‐ZAR1 complex (ADP free) revealed effector‐triggered allosteric ADP release as a key step to prime NLR for ATP binding and activation (Wang et al., 2019b) (Figure 2e).

## 4 N‐TERMINAL SIGNALLING DOMAIN: DEFINING THE BIOCHEMICAL FUNCTIONS OF NLRS

The N‐terminal TIR, CC, or CC^R^ domains of NLRs are the signalling domains, overexpression of which in *N. benthamiana* is sufficient to trigger cell death (Bernoux et al., 2011; Collier et al., 2011; Maekawa et al., 2011; Swiderski et al., 2009). Structural biology studies on plant NLRs were limited to the N‐terminal signalling domains before the advancement of cryo‐EM as a technique to solve full‐length NLR structures.

## 5 UNEXPECTED ENZYMATIC ACTIVITY OF TIR DOMAINS

**FIGURE 3:**
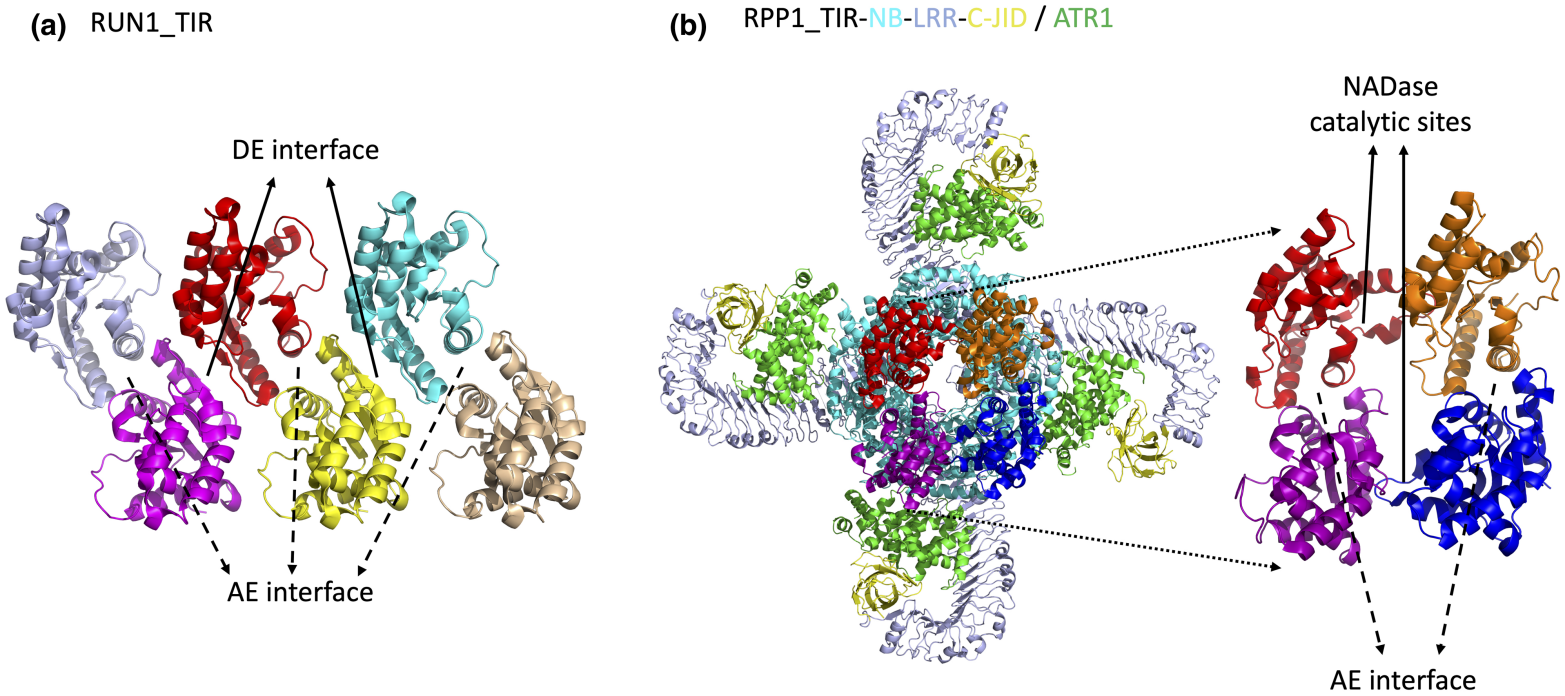
Plant TIR dimerization interfaces and higher oligomer formation. (a) Crystal structure of the RUN1 TIR domain highlighting filament formation through continuation of alternating AE and DE interfaces. (b) Structure of RPP1 tetrameric resistosome highlighting the TIR tetramer including two symmetrical AE dimerization interfaces and two asymmetric interfaces as NADase catalytic sites

Crystal structures of a few plant TNL TIR domains have been reported, including *Arabidopsis* RPS4, RRS1, SNC1 (suppressor of npr1‐1, constitutive 1), RPP1, flax L6, and grape RUN1 (Resistance to *Uncinula necator* 1) (Bernoux et al., 2011; Horsefield et al., 2019; Williams et al., 2014; Zhang et al., 2017a). Plant TIR domains feature a global fold consisting of a five‐stranded parallel β sheet (βA–βE) surrounded by five α‐helical regions (αA–αE). The global fold is similar to animal and bacterial TIR structures. The signalling incompetent TIR domain of RRS1 functioning as a sensor in the RPS4/RRS1 pair system harbours a deletion in the αD helical region compared to the signalling competent TIR domain of RPS4 functioning as an immune executor (Williams et al., 2014). A well‐characterized dimerization interface identified from the crystal contacts is the one mainly involving the αA and αE helices with a conserved serine and histidine motif at the core of this AE interface (Williams et al., 2014). Another dimerization interface mainly involving the αD and αE helices, termed the DE interface, is positioned in slightly different orientations in different TIR crystal structures and thus is less well defined (Zhang et al., 2017a). TIR self‐association via the AE and DE interfaces is required for the cell death function of plant TIR domains. AE and DE interfaces are not mutually exclusively and could potentially mediate the formation of TIR filament structures (Nishimura et al., 2017; Zhang et al., 2017a) (Figure 3a). Animal TIR proteins have been shown to form filament structures in vitro (Ve et al., 2017).

Structures of plant TIRs and their dimeric forms do not implicate the biochemical functions. Well‐characterized TIR domains of animal cell‐surface toll‐like receptors dimerize on activation to recruit downstream signalling proteins (Fitzgerald & Kagan, 2020). Hence scientists thought plant TIRs would also function as scaffold proteins. In 2017 and 2018, some bacterial TIR domains and the TIR domain of animal SARM1 involved in the cell death pathway of neuron degeneration were demonstrated to possess enzymatic activity, cleaving nicotinamide adenine dinucleotide (NAD^+^) (Essuman et al., 2017, 2018). Follow‐up investigations confirmed that plant TIRs indeed function as NADase to trigger cell death dependent on the conserved catalytical glutamate and the integrity of the two dimerization interfaces (Horsefield et al., 2019; Wan et al., 2019). Different from the more active SARM1 enzyme that causes NAD^+^ depletion and subsequent cell death, plant TIRs domains are weak NADase without causing NAD^+^ depletion and potentially produce signalling molecules such as nicotinamide (NAM), adenosine diphosphate ribose (ADPR), and variant‐cADPR (an isomer of cyclic ADPR) to bind and activate proteins downstream (Wan et al., 2019).

## 6 CONSERVED FOUR‐HELICAL‐BUNDLE FOLD OF CC AND CC^R^ DOMAINS FOR CELL DEATH FUNCTION

**FIGURE 4:**
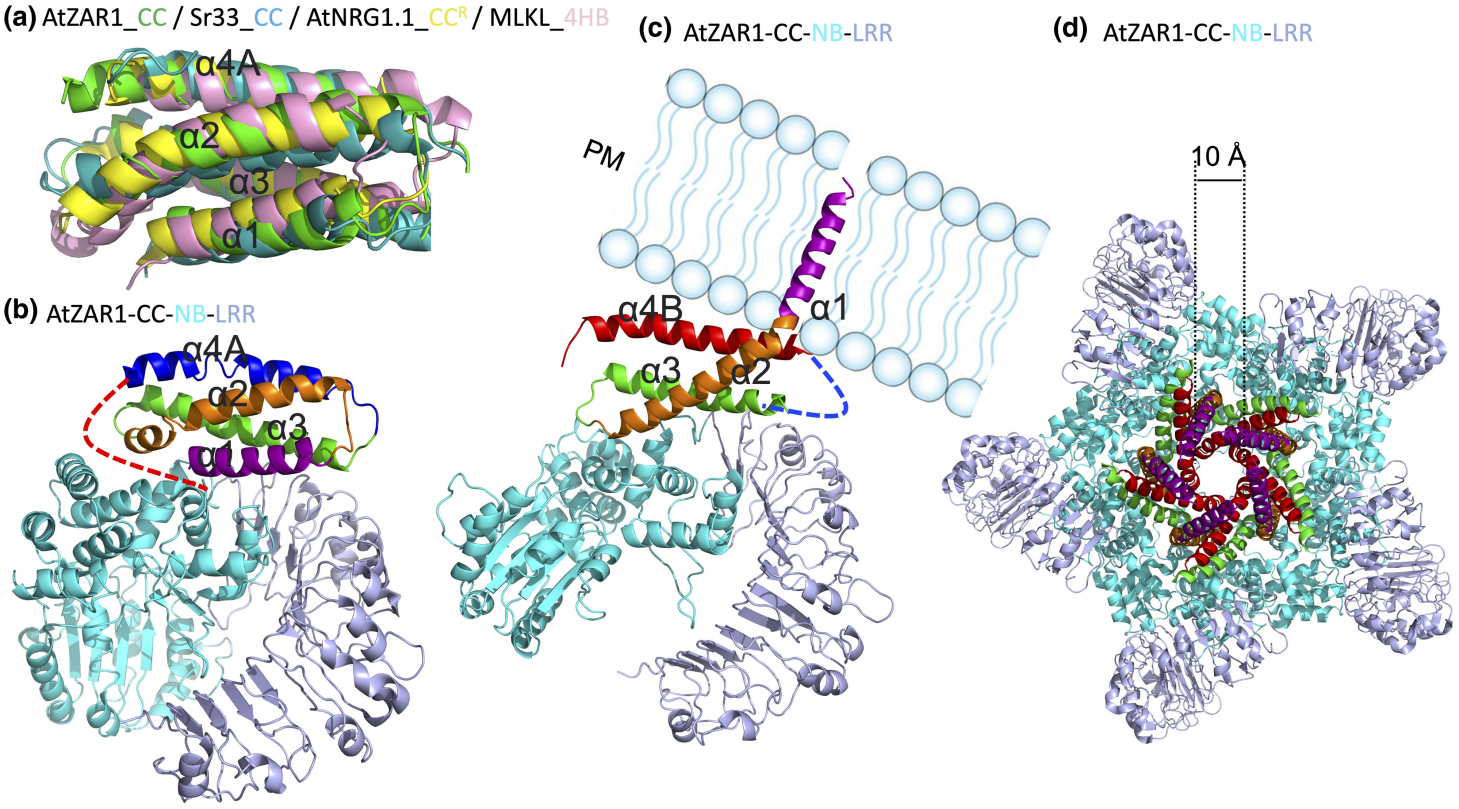
Conserved four‐helical‐bundle (4HB) fold structure of plant NLR CC and CC^R^ domains, and the dynamics of ZAR1 resistosome formation. (a) Superimposition of AtZAR1 CC in green (PDB: 6J5W), Sr33 CC in teal (PDB: 2NCG), AtNRG1.1 CC^R^ in yellow (PDB: 7L7W), and mouse MLKL 4HB in pink (PDB: 4BTF). (b) Structure of AtZAR1 (PDB: 6J5W) in the resting state. The four helices of the CC domain are coloured in purple, orange, green, and blue. The loop region connecting α4A and NB is shown as a curved dashed line in red, which becomes a helix termed α4B in the active state. (c) Structure of active AtZAR1 (PDB: 6J5T) shown as a monomer from the pentameric resistosome highlighting the α1 helix being flipped and penetrating the plasma membrane (PM). The α4A in the resting state becomes a potentially flexible loop region not resolved in the electron density map and is indicated as a curved dashed line in blue. The newly formed helix α4B is shown in red. (d) Structure of AtZAR1 resistosome (PDB: 6J5T) highlighting the channel pore with a diameter of about 1 nm

A few NLR CC structures have been reported, including barley MLA10, potato Rx, and wheat Sr33, which collectively suggest that the CC domain (amino acids 1–120) adopts a conserved four‐helical‐bundle (4HB) fold as a monomer (Case y et al., 2016; Hao et al., 2013). A longer fragment, for instance amino acids 1–144 in Sr33, is the minimum to self‐associate and trigger cell death when overexpressed in *N. benthamiana*, indicating the functional importance of sequences located C‐terminally to the 4HB domain (Cesari et al., 2016). The CC^R^ domains of NRG1, ADR1, and RPW8 share low sequence similarity of about 10% with the canonical CC domains of CNLs. Structure modelling of ADR1 and NRG1 CC^R^ domains also suggested a potential 4HB fold and relevance to other 4HB‐containing cell death factors of membrane pore‐forming proteins, such as animal Mixed Lineage Kinase Domain‐Like (MLKL) protein and fungal HeLo and HeLo‐like proteins (Bentham et al., 2018; Daskalov et al., 2016; Jubic et al., 2019; Mahdi et al., 2020). The crystal structure of AtNRG1.1 CC^R^ confirmed the 4HB fold of CC^R^ (Jacob et al., 2021) (Figure 4a). AtNRG1.1 CC^R^ harbours an extra region N‐terminal to the 4HB fold compared to canonical CC domains, which is consistent with previous structural predictions of CC^R^ and RPW8. The extra region N‐terminal to the 4HB fold of CC^R^ and RPW8 is predicted to be responsible for membrane anchoring (Zhong & Cheng, 2016). A recent study showed that deletion of the extra region N‐terminal to the 4HB fold impairs the cell death function of auto‐active AtNRG1.1 but not its membrane localization (Jacob et al., 2021). It has also been shown that RNLs interact with anionic plasma membrane (PM) phospholipids, and depletion of phosphatidylinositol‐4‐phosphate from the PM causes mislocalization of RNLs and abolishes their cell death activities (Saile et al., 2021).

MLKL is a well‐studied membrane pore‐forming protein (Murphy et al., 2013). Current modelling suggests that MLKL remains cytosolic and monomeric in the resting state, but starts to oligomerize, move to the PM, and form pores at the PM, leading to loss of membrane integrity and cell death (Huang et al., 2017). The exact oligomerization state and pore size of activated MLKL are still unknown. It has also been shown that MLKL functions as a cation channel to induce cell death, implicating the possible channel activities of plant CNLs and RNLs (Xia et al., 2016).

## 7 NLR RESISTOSOMES AND SIGNALLING

Self‐association is known to be required for NLR function. The NB domain is responsible for the self‐association and oligomerization of NLRs and functions as a molecular switch bridging immune stimulus perception by the LRR domain and immune signalling by the N‐terminal domain (Burdett et al., 2019; Saur et al., 2021).

## 8 CNL ZAR1 PENTAMERIC RESISTOSOME FUNCTIONING AS A Ca^2+^ CHANNEL

In 2019, cryo‐EM strudies of full‐length ZAR1 in both resting and active states for the very first time revealed the structure of interdomain interactions for autoinhibition and oligomerization for immune activation of a plant NLR (Wang et al., 2019a, 2019b). The structure of monomeric ADP‐bound ZAR1 in complex with RKS1 represents the resting state. RKS1 interacts exclusively with the ZAR1 LRR domain to maintain ZAR1 in the resting state (Wang et al., 2019b). The structure of dATP‐bound ZAR1 in complex with RKS1 and effector‐produced PBL2^UMP^ represents the active state. On effector recognition, ZAR1 undergoes dramatic conformational changes from an inactive monomeric state to an active pentameric state with the first helix in the 4HB fold of its CC domain being flipped, potentially forming a pore at the PM. In addition to the first helix α1 being flipped, the fourth helix α4A becomes disordered in the active state. Previously identified sequences C‐terminal to the 4HB domain that are critical for self‐association and cell death function are largely disordered in the resting state but become a helix, termed α4B, in the active state (Figure 4b,c). These conformational changes in the CC domain and pentamerization driven by the NB domain feature the dynamics of ZAR1 resistosome formation. The ZAR1 resistosome displays a channel pore of 1 nm in diameter (Wang et al., 2019a) (Figure 4d). A follow‐up study confirmed that the effector‐activated ZAR1 resistosome protrudes into the PM and forms a calcium‐permeable cation channel leading to calcium influx and further activation of cell death and defence responses (Bi et al., 2021) (Figure S1a).

## 9 TNL RPP1 AND ROQ1 TETRAMERIC RESISTOSOMES FUNCTIONING AS NADase

Recent cryo‐EM strudies of TNL/effector pairs ROQ1/XopQ and RPP1/ATR1 structures, followed by structure‐guided biochemical studies in vitro and functional analyses in planta, greatly advanced our understanding of TNL activation mechanisms and functioning as a NAD^+^ cleavage enzyme (Ma et al., 2020; Martin et al., 2020). On effector recognition, ROQ1 and RPP1 assemble into homotetrameric resistosomes. An apparent difference is that the ROQ1 resistosome binds an ATP molecule in the NB domain as expected, while the RPP1 resistosome binds an ADP molecule although ATP was supplemented in the purification process. Further analyses indicated that plant CNLs and some TNLs, such as ROQ1, use a “TT/SR” motif for ATP binding, while some TNLs, such as RPP1, have a “TTE/Q” motif, losing the ATP binding ability and bind ADP molecules (Ma et al., 2020). Tetramerization in the NB domain drives the formation of the TIR tetramer as an active holoenzyme. The TIR tetramer contains two previously characterized symmetrical AE interfaces and two asymmetric interfaces that create the potential active sites for NAD^+^ cleavage catalysis (Figure 3b). Interestingly, each of the two active sites in the RPP1 resistosome binds an ATP molecule, which was supplemented during purification. The bound ATP probably acts as an analogue of NAD^+^ at the active site. More interestingly, structural analyses revealed that NADP^+^ binds to the TIR domain of RUN1 at a similar position to the ATP‐occupied active site (Horsefield et al., 2019; Ma et al., 2020). These observations support the idea that active TNL resistosomes function as a holoenzyme cleaving NAD(P)^+^ to transduce the signal in the absence of NAD(P)^+^‐bound TNL resistosome structures. However, the identity of the TNL‐produced signalling molecules remains elusive.

## 10 NUCLEASE ACTIVITIES OF PLANT TIR FILAMENTS

Previous crystal structures of plant TIR domains identified two dimerization interfaces required for function (Zhang et al., 2017a). The functional relevance of the AE dimerization interface was demonstrated in TNL tetrameric resistosomes for NADase activity. However, the DE interface was not observed in TNL tetrameric resistosomes and its functional relevance remains unclear. Interestingly, a recent study showed that the TIR domain of flax TNL L7 can assemble into a filament using the AE and DE interfaces, as proposed previously (Figure 3a). Moreover, the L7 TIR domain was purified in complex with DNA. Cryo‐EM studies of the structure of the L7 TIR filament wrapping DNA showed that filament–DNA interaction is critical for TIR nuclease activity and production of 2′,3′‐cAMP (Yu et al., 2021). 2′,3′‐cAMP molecules were identified in the NAD^+^‐binding pocket of L7 TIR domains in the TIR filament structure, suggesting that NADase and nuclease activities of TIR domains use similar catalytical sites. Further mutational studies confirmed that 2′,3′‐cAMP synthetase activity correlates with TIR cell death function (Yu et al., 2021). It is not clear whether 2′,3′‐cAMP functions as a signalling molecule. Thus, plant TIR domains could assemble into filaments and function as nucleases, but the possibility of TIR filament formation in the context of full‐length TNLs and the distinct roles of TIR NADase and nuclease activities in TIR signalling remain unknown.

## 11 UNDEFINED FUNCTIONS OF LIPASE‐LIKE PROTEINS AND RNLS DOWNSTREAM OF TNL

All tested TNLs require lipase‐like proteins, including Enhanced Disease Susceptibility1 (EDS1), Senescence‐Associated Gene101 (SAG101), and Phytoalexin Deficient4 (PAD4), for function (Gantner et al., 2019; Lapin et al., 2019; Wagner et al., 2013). In *Arabidopsis*, AtEDS1 and AtSAG101 form a stable heterodimer and cooperate with the AtNRG1s to control cell death, while AtEDS1 and AtPAD4 physically associate and function together with the AtADR1s to mediate bacterial growth restriction and resistance. EDS1, SAG101, and PAD4 contain a lipase‐like domain, whose enzymatic activity is dispensable for function, and a plant‐unique EDS1‐PAD4 (EP) domain. In EDS1/SAG101 and EDS1/PAD4 heterodimers, the pockets between the two EP domains are critical for TNL function and are proposed to be the potential binding sites of TIR‐generated small molecules, but are not validated (Sun et al., 2021). More interestingly, activation of the TIR signalling pathway has been shown to trigger the association between EDS1/SAG101and NRG1s as well as the interaction between EDS1/PAD4 and ADR1s (Sun et al., 2021; Wu et al., 2021). The activation mechanisms of EDS1/SAG101/NRG1 and EDS1/PAD4/ADR1 to initiate cell death and resistance responses, respectively, are currently unknown.

## 12 AUTOACTIVE NRG1 AND ADR1 FUNCTION AS A Ca^2+^ CHANNEL

Similar to lipase‐like proteins, RNLs including NRG1s and ADR1s are genetic requirements for TNL function (Castel et al., 2019; Qi et al., 2018; Saile et al., 2020). In the context of ETI, RNLs function cooperatively with lipase‐like proteins. Autoactive RNLs or their CC^R^ domains trigger EDS1‐independent cell death in *N. benthamiana* (Collier et al., 2011; Jacob et al., 2021). The AtNRG1.1 CC^R^ domain adopts a 4HB structure fold reminiscent of the cell death domain of MLKL that has been shown to function as cation channel. Further investigations demonstrated that autoactive NRG1 mutant and ADR1 function as a Ca^2+^ channel when expressed in planta and in human cells to trigger cell death (Jacob et al., 2021) (Figure S2a).

## 13 NLR SIGNALLING NETWORK AND UNRESOLVED LINKS

Based on current knowledge, a plant NLR functioning network includes three distinct signalling modes. First, some CNL singletons like ZAR1 are both sensors of pathogen effectors and immunity executors as a Ca^2+^ channel (Figure S1a). Whether all CNL singletons and pairs function as Ca^2+^ channels or alternative signalling mechanisms exist remains to be demonstrated. The structural bases of CNL pairs autoinhibition and activation require future investigations. Second, in solanaceous species, phylogenetically related sensor CNLs on effector recognition activate CNL‐type helper NRCs to confer resistance and cell death. Interestingly, the pore‐forming helix α1 in the CC domain is interchangeable between ZAR1 and NRCs. It is reasonable to speculate that helper NRCs function as Ca^2+^ channel‐like ZAR1 and RNL helpers, which is not confirmed experimentally. The biochemical functions of the sensor CNLs and how effector‐activated sensor CNLs further activate helper NRCs are not clear. Whether sensor CNLs are also Ca^2+^ channels or use different biochemical function to activate NRCs remains to be determined (Figure S1b). Third, effector‐activated TNLs function as NADase and nuclease to produce undefined signalling molecules whose receptors remain to be determined. Whether full‐length TNLs can form filaments and function as nucleases remains to be demonstrated. The activation mechanisms of EDS1/SAG101/NRG1 and EDS1/PAD4/ADR1 by two different TIR enzymatic activities are not clear. The biochemical function of lipase‐like family proteins EDS1/SAG101/PAD4 in the TNL signalling pathway is not clear either. In the biologically relevant context of the effector‐activated TNL signalling pathway involving EDS1/SAG101 and EDS1/PAD4, whether RNLs indeed function as a Ca^2+^ channel requires further investigation (Figure S2b). The structural bases of helper RNLs functioning as a Ca^2+^ channel remain unresolved. The structures of TNL pairs in the resting and active states are not known either. The Ca^2+^‐responsive factors perceiving the channel activities of CNLs, RNLs, and potential NRCs and their links to immune signalling require further investigation.

## Supporting information

Figure S1 CNL activation and signalling pathways. (a) AtZAR1 functions as an NLR singleton that does both jobs of sensor NLR and helper NLR to confer immunity and cause cell death. (b) Sensor CNLs on effector recognition activate immunity and cell death through NRC helpers that potentially function as Ca^2+^ channels

Figure S2 TNL activation and signalling pathways. (a) Autoactive NRG1 and ADR1 function as Ca^2+^ channels to induce cell death. (b) TNL and TIR‐only receptors function as enzymes to produce small signalling molecules that activate complexes of EDS1/SAG101/NRG1 and EDS1/PAD4/ADR1, and potentially lead to NRG1 and ADR1 Ca^2+^ channel formation

## Data Availability

Data sharing is not applicable to this article as no new data were created or analysed.

## References

[mpp13195-bib-0001] Adachi, H. , Derevnina, L. & Kamoun, S. (2019) NLR singletons, pairs, and networks: evolution, assembly, and regulation of the intracellular immunoreceptor circuitry of plants. Current Opinion in Plant Biology, 50, 121–131. 31154077 10.1016/j.pbi.2019.04.007

[mpp13195-bib-0002] Adachi, H. , Sakai, T. , Harant, A. , Duggan, C. , Bozkurt, C. , Wu, C. & Kamoun, S. (2021) An atypical NLR protein modulates the NRC immune receptor network. bioRxiv. 10.1101/2021.11.15.468391 PMC9851556 36656829

[mpp13195-bib-0003] Bentham, A.R. , Zdrzalek, R. , De la Concepcion, J.C. & Banfield, M.J. (2018) Uncoiling CNLs: structure/function approaches to understanding CC domain function in plant NLRs. Plant and Cell Physiology, 59, 2398–2408. 30192967 10.1093/pcp/pcy185 PMC6290485

[mpp13195-bib-0004] Bernoux, M. , Ve, T. , Williams, S. , Warren, C. , Hatters, D. , Valkov, E. et al. (2011) Structural and functional analysis of a plant resistance protein TIR domain reveals interfaces for self‐association, signaling, and autoregulation. Cell Host & Microbe, 9, 200–211. 21402359 10.1016/j.chom.2011.02.009 PMC3142617

[mpp13195-bib-0005] Bi, G. , Su, M. , Li, N. , Liang, Y.U. , Dang, S. , Xu, J. et al. (2021) The ZAR1 resistosome is a calcium‐permeable channel triggering plant immune signaling. Cell, 184, 3528–3541. 33984278 10.1016/j.cell.2021.05.003

[mpp13195-bib-0006] Bialas, A. , Langner, T. , Harant, A. , Contreras, M.P. , Stevenson, C.E.M. , Lawson, D.M. et al. (2021) Two NLR immune receptors acquired high‐affinity binding to a fungal effector through convergent evolution of their integrated domain. eLife, 10, e66961. 34288868 10.7554/eLife.66961 PMC8294853

[mpp13195-bib-0007] Burdett, H. , Bentham, A.R. , Williams, S.J. , Dodds, P.N. , Anderson, P.A. , Banfield, M.J. et al. (2019) The plant “resistosome”: structural insights into immune signaling. Cell Host & Microbe, 26, 193–201. 31415752 10.1016/j.chom.2019.07.020

[mpp13195-bib-0008] Casey, L.W. , Lavrencic, P. , Bentham, A.R. , Cesari, S. , Ericsson, D.J. , Croll, T. et al. (2016) The CC domain structure from the wheat stem rust resistance protein Sr33 challenges paradigms for dimerization in plant NLR proteins. Proceedings of the National Academy of Sciences of the United States of America, 113, 12856–12861. 27791121 10.1073/pnas.1609922113 PMC5111715

[mpp13195-bib-0009] Castel, B. , Ngou, P.M. , Cevik, V. , Redkar, A. , Kim, D.S. , Yang, Y. et al. (2019) Diverse NLR immune receptors activate defence via the RPW8‐NLR NRG1. New Phytologist, 222, 966–980. 30582759 10.1111/nph.15659

[mpp13195-bib-0010] Cesari, S. , Moore, J. , Chen, C. , Webb, D. , Periyannan, S. , Mago, R. et al. (2016) Cytosolic activation of cell death and stem rust resistance by cereal MLA‐family CC‐NLR proteins. Proceedings of the National Academy of Sciences of the United States of America, 113, 10204–10209. 27555587 10.1073/pnas.1605483113 PMC5018743

[mpp13195-bib-0011] Cesari, S. , Thilliez, G. , Ribot, C. , Chalvon, V. , Michel, C. , Jauneau, A. et al. (2013) The rice resistance protein pair RGA4/RGA5 recognizes the *Magnaporthe oryzae* effectors AVR‐Pia and AVR1‐CO39 by direct binding. The Plant Cell, 25, 1463–1481. 23548743 10.1105/tpc.112.107201 PMC3663280

[mpp13195-bib-0012] Collier, S.M. , Hamel, L.P. & Moffett, P. (2011) Cell death mediated by the N‐terminal domains of a unique and highly conserved class of NB‐LRR protein. Molecular Plant‐Microbe Interactions, 24, 918–931. 21501087 10.1094/MPMI-03-11-0050

[mpp13195-bib-0013] Daskalov, A. , Habenstein, B. , Sabaté, R. , Berbon, M. , Martinez, D. , Chaignepain, S. et al. (2016) Identification of a novel cell death‐inducing domain reveals that fungal amyloid‐controlled programmed cell death is related to necroptosis. Proceedings of the National Academy of Sciences of the United States of America, 113, 2720–2725. 26903619 10.1073/pnas.1522361113 PMC4790977

[mpp13195-bib-0014] De la Concepcion, J.C. , Franceschetti, M. , MacLean, D. , Terauchi, R. , Kamoun, S. & Banfield, M.J. (2019) Protein engineering expands the effector recognition profile of a rice NLR immune receptor. eLife, 8, e47713. 31535976 10.7554/eLife.47713 PMC6768660

[mpp13195-bib-0015] De la Concepcion, J.C. , Franceschetti, M. , Maqbool, A. , Saitoh, H. , Terauchi, R. , Kamoun, S. et al. (2018) Polymorphic residues in rice NLRs expand binding and response to effectors of the blast pathogen. Nature Plants, 4, 576–585. 29988155 10.1038/s41477-018-0194-x

[mpp13195-bib-0016] DeFalco, T.A. & Zipfel, C. (2021) Molecular mechanisms of early plant pattern‐triggered immune signaling. Molecular Cell, 81, 3449–3467. 34403694 10.1016/j.molcel.2021.07.029

[mpp13195-bib-0017] Duxbury, Z. , Wu, C.H. & Ding, P.T. (2021) A Comparative overview of the intracellular guardians of plants and animals: NLRs in innate immunity and beyond. Annual Review of Plant Biology, 72, 155–184. 10.1146/annurev-arplant-080620-104948 33689400

[mpp13195-bib-0018] Essuman, K. , Summers, D.W. , Sasaki, Y. , Mao, X.R. , DiAntonio, A. & Milbrandt, J. (2017) The SARM1 toll/interleukin‐1 receptor domain possesses intrinsic NAD(+) cleavage activity that promotes pathological axonal degeneration. Neuron, 93, 1334–1343. 28334607 10.1016/j.neuron.2017.02.022 PMC6284238

[mpp13195-bib-0019] Essuman, K. , Summers, D.W. , Sasaki, Y.O. , Mao, X. , Yim, A.K.Y. , DiAntonio, A. et al. (2018) TIR domain proteins are an ancient family of NAD(+)‐consuming enzymes. Current Biology, 28, 421–430. 29395922 10.1016/j.cub.2017.12.024 PMC5802418

[mpp13195-bib-0020] Fitzgerald, K.A. & Kagan, J.C. (2020) Toll‐like receptors and the control of immunity. Cell, 180, 1044–1066. 32164908 10.1016/j.cell.2020.02.041 PMC9358771

[mpp13195-bib-0021] Gantner, J. , Ordon, J. , Kretschmer, C. , Guerois, R. & Stuttmann, J. (2019) An EDS1‐SAG101 complex is essential for TNL‐mediated immunity in *Nicotiana benthamiana* . The Plant Cell, 31, 2456–2474. 31266900 10.1105/tpc.19.00099 PMC6790086

[mpp13195-bib-0022] Hao, W. , Collier, S.M. , Moffett, P. & Chai, J. (2013) Structural basis for the interaction between the potato virus X resistance protein (Rx) and its cofactor Ran GTPase‐activating protein 2 (RanGAP2). Journal of Biological Chemistry, 288, 35868–35876. 24194517 10.1074/jbc.M113.517417 PMC3861636

[mpp13195-bib-0023] Horsefield, S. , Burdett, H. , Zhang, X. , Manik, M.K. , Shi, Y. , Chen, J. et al. (2019) NAD(+) cleavage activity by animal and plant TIR domains in cell death pathways. Science, 365, 793–799. 31439792 10.1126/science.aax1911

[mpp13195-bib-0024] Huang, D. , Zheng, X. , Wang, Z.‐A. , Chen, X. , He, W.‐T. , Zhang, Y. et al. (2017) The MLKL channel in necroptosis is an octamer formed by tetramers in a dyadic process. Molecular and Cellular Biology, 37, e00497‐16. 27920255 10.1128/MCB.00497-16 PMC5311246

[mpp13195-bib-0025] Jacob, P. , Kim, N.H. , Wu, F.H. , El Kasmr, F. , Chi, Y. , Walton, W.G. et al. (2021) Plant “helper” immune receptors are Ca^2+^‐permeable nonselective cation channels. Science, 373, 420–425. 34140391 10.1126/science.abg7917 PMC8939002

[mpp13195-bib-0026] Jones, J.D.G. , Vance, R.E. & Dangl, J.L. (2016) Intracellular innate immune surveillance devices in plants and animals. Science, 354, aaf6395. 27934708 10.1126/science.aaf6395

[mpp13195-bib-0027] Jubic, L.M. , Saile, S. , Furzer, O.J. , El Kasmi, F. & Dangl, J.L. (2019) Help wanted: helper NLRs and plant immune responses. Current Opinion in Plant Biology, 50, 82–94. 31063902 10.1016/j.pbi.2019.03.013

[mpp13195-bib-0028] Lapin, D. , Kovacova, V. , Sun, X. , Dongus, J.A. , Bhandari, D. , von Born, P. et al. (2019) A coevolved EDS1‐SAG101‐NRG1 module mediates cell death signaling by TIR‐domain immune receptors. The Plant Cell, 31, 2430–2455. 31311833 10.1105/tpc.19.00118 PMC6790079

[mpp13195-bib-0029] Le Roux, C. , Huet, G. , Jauneau, A. , Camborde, L. , Trémousaygue, D. , Kraut, A. et al. (2015) A receptor pair with an integrated decoy converts pathogen disabling of transcription factors to immunity. Cell, 161, 1074–1088. 26000483 10.1016/j.cell.2015.04.025

[mpp13195-bib-0030] Ma, S. , Lapin, D. , Liu, L.I. , Sun, Y. , Song, W. , Zhang, X. et al. (2020) Direct pathogen‐induced assembly of an NLR immune receptor complex to form a holoenzyme. Science, 370, eabe3069. 33273071 10.1126/science.abe3069

[mpp13195-bib-0031] Maekawa, T. , Cheng, W. , Spiridon, L.N. , Töller, A. , Lukasik, E. , Saijo, Y. et al. (2011) Coiled‐coil domain‐dependent homodimerization of intracellular barley immune receptors defines a minimal functional module for triggering cell death. Cell Host & Microbe, 9, 187–199. 21402358 10.1016/j.chom.2011.02.008

[mpp13195-bib-0032] Mahdi, L.K. , Huang, M. , Zhang, X. , Nakano, R.T. , Kopp, L.B. , Saur, I.M.L. et al. (2020) Discovery of a family of mixed lineage kinase domain‐like proteins in plants and their role in innate immune signaling. Cell Host & Microbe, 28, 813–824. 33053377 10.1016/j.chom.2020.08.012

[mpp13195-bib-0033] Martin, R. , Qi, T. , Zhang, H. , Liu, F. , King, M. , Toth, C. et al. (2020) Structure of the activated ROQ1 resistosome directly recognizing the pathogen effector XopQ. Science, 370, eabd9993. 33273074 10.1126/science.abd9993 PMC7995448

[mpp13195-bib-0034] Mukhi, N. , Brown, H. , Gorenkin, D. , Ding, P.T. , Bentham, A. , Stevenson, C. , Jones, J.D.G. & Banfield, M.J. (2021) Perception of structurally distinct effectors by the integrated WRKY domain of a plant immune receptor. Proceedings of the National Academy of Sciences of the Unites States of America, 118, e2113996118. 10.1073/pnas.2113996118 PMC8685902 34880132

[mpp13195-bib-0035] Murphy, J. , Czabotar, P. , Hildebrand, J. , Lucet, I. , Zhang, J.‐G. , Alvarez‐Diaz, S. et al. (2013) The pseudokinase MLKL mediates necroptosis via a molecular switch mechanism. Immunity, 39, 443–453. 24012422 10.1016/j.immuni.2013.06.018

[mpp13195-bib-0036] Narusaka, M. , Shirasu, K. , Noutoshi, Y. , Kubo, Y. , Shiraishi, T. , Iwabuchi, M. et al. (2009) RRS1 and RPS4 provide a dual resistance‐gene system against fungal and bacterial pathogens. The Plant Journal, 60, 218–226. 19519800 10.1111/j.1365-313X.2009.03949.x

[mpp13195-bib-0037] Ngou, B.P.M. , Ahn, H.K. , Ding, P.T. & Jones, J.D.G. (2021) Mutual potentiation of plant immunity by cell‐surface and intracellular receptors. Nature, 592, 110–115. 33692545 10.1038/s41586-021-03315-7

[mpp13195-bib-0038] Nishimura, M.T. , Anderson, R.G. , Cherkis, K.A. , Law, T.F. , Liu, Q.L. , Machius, M. et al. (2017) TIR‐only protein RBA1 recognizes a pathogen effector to regulate cell death in Arabidopsis. Proceedings of the National Academy of Sciences of the United States of America, 114, E2053–E2062. 28137883 10.1073/pnas.1620973114 PMC5347586

[mpp13195-bib-0039] Pruitt, R.N. , Locci, F. , Wanke, F. , Zhang, L. , Saile, S.C. , Joe, A. et al. (2021) The EDS1‐PAD4‐ADR1 node mediates *Arabidopsis* pattern‐triggered immunity. Nature, 598, 495–499. 34497423 10.1038/s41586-021-03829-0

[mpp13195-bib-0040] Qi, T. , Seong, K. , Thomazella, D.P.T. , Kim, J.R. , Pham, J. , Seo, E. et al. (2018) NRG1 functions downstream of EDS1 to regulate TIR‐NLR‐mediated plant immunity in *Nicotiana benthamiana* . Proceedings of the National Academy of Sciences of the United States of America, 115, E10979–E10987. 30373842 10.1073/pnas.1814856115 PMC6243234

[mpp13195-bib-0041] Saile, S.C. , Ackermann, F.M. , Sunil, S. , Keicher, J. , Bayless, A. , Bonardi, V. et al. (2021) Arabidopsis ADR1 helper NLR immune receptors localize and function at the plasma membrane in a phospholipid dependent manner. New Phytologist, 232, 2440–2456. 34628646 10.1111/nph.17788

[mpp13195-bib-0042] Saile, S.C. , Jacob, P. , Castel, B. , Jubic, L.M. , Salas‐Gonzales, I. , Backer, M. et al. (2020) Two unequally redundant “helper” immune receptor families mediate *Arabidopsis thaliana* intracellular “sensor” immune receptor functions. PLoS Biology, 18, e3000783. 32925907 10.1371/journal.pbio.3000783 PMC7514072

[mpp13195-bib-0043] Saur, I.M.L. , Panstruga, R. & Schulze‐Lefert, P. (2021) NOD‐like receptor‐mediated plant immunity: from structure to cell death. Nature Reviews Immunology, 21, 305–318. 10.1038/s41577-020-00473-z 33293618

[mpp13195-bib-0044] Sun, X. , Lapin, D. , Feehan, J.M. , Stolze, S.C. , Kramer, K. , Dongus, J.A. et al. (2021) Pathogen effector recognition‐dependent association of NRG1 with EDS1 and SAG101 in TNL receptor immunity. Nature Communications, 12, 3335. 10.1038/s41467-021-23614-x PMC8185089 34099661

[mpp13195-bib-0045] Swiderski, M.R. , Birker, D. & Jones, J.D. (2009) The TIR domain of TIR‐NB‐LRR resistance proteins is a signaling domain involved in cell death induction. Molecular Plant‐Microbe Interactions, 22, 157–165. 19132868 10.1094/MPMI-22-2-0157

[mpp13195-bib-0046] Tian, H. , Wu, Z. , Chen, S. , Ao, K. , Huang, W. , Yaghmaiean, H. et al. (2021) Activation of TIR signalling boosts pattern‐triggered immunity. Nature, 598, 500–503. 34544113 10.1038/s41586-021-03987-1

[mpp13195-bib-0047] Ve, T. , Vajjhala, P.R. , Hedger, A. , Croll, T. , DiMaio, F. , Horsefield, S. et al. (2017) Structural basis of TIR‐domain‐assembly formation in MAL‐ and MyD88‐dependent TLR4 signaling. Nature Structural & Molecular Biology, 24, 743–751. 10.1038/nsmb.3444 PMC8059215 28759049

[mpp13195-bib-0048] Wagner, S. , Stuttmann, J. , Rietz, S. , Guerois, R. , Brunstein, E. , Bautor, J. et al. (2013) Structural basis for signaling by exclusive EDS1 heteromeric complexes with SAG101 or PAD4 in plant innate immunity. Cell Host & Microbe, 14, 619–630. 24331460 10.1016/j.chom.2013.11.006

[mpp13195-bib-0049] Wan, L. , Essuman, K. , Anderson, R.G. , Sasaki, Y. , Monteiro, F. , Chung, E.H. et al. (2019) TIR domains of plant immune receptors are NAD(+)‐cleaving enzymes that promote cell death. Science, 365, 799–803. 31439793 10.1126/science.aax1771 PMC7045805

[mpp13195-bib-0050] Wang, G. , Roux, B. , Feng, F. , Guy, E. , Li, L. , Li, N. et al. (2015) The decoy substrate of a pathogen effector and a pseudokinase specify pathogen‐induced modified‐self recognition and immunity in plants. Cell Host & Microbe, 18, 285–295. 26355215 10.1016/j.chom.2015.08.004

[mpp13195-bib-0051] Wang, J. , Hu, M. , Wang, J. , Qi, J. , Han, Z. , Wang, G. et al. (2019a) Reconstitution and structure of a plant NLR resistosome conferring immunity. Science, 364, eaav5870. 30948527 10.1126/science.aav5870

[mpp13195-bib-0052] Wang, J. , Wang, J. , Hu, M. , Wu, S. , Qi, J. , Wang, G. et al. (2019b) Ligand‐triggered allosteric ADP release primes a plant NLR complex. Science, 364, eaav5868. 30948526 10.1126/science.aav5868

[mpp13195-bib-0053] Wang, W. , Feng, B.M. , Zhou, J.M. & Tang, D.Z. (2020) Plant immune signaling: advancing on two frontiers. Journal of Integrative Plant Biology, 62, 2–24. 31846204 10.1111/jipb.12898

[mpp13195-bib-0054] Williams, S.J. , Sohn, K.H. , Wan, L.I. , Bernoux, M. , Sarris, P.F. , Segonzac, C. et al. (2014) Structural basis for assembly and function of a heterodimeric plant immune receptor. Science, 344, 299–303. 24744375 10.1126/science.1247357

[mpp13195-bib-0055] Wu, C.‐H. , Abd‐El‐Haliem, A. , Bozkurt, T.O. , Belhaj, K. , Terauchi, R. , Vossen, J.H. et al. (2017) NLR network mediates immunity to diverse plant pathogens. Proceedings of the National Academy of Sciences of the United States of America, 114, 8113–8118. 28698366 10.1073/pnas.1702041114 PMC5544293

[mpp13195-bib-0056] Wu, Z.S. , Tian, L. , Liu, X.R. , Zhang, Y.L. & Li, X. (2021) TIR signal promotes interactions between lipase‐like proteins and ADR1‐L1 receptor and ADR1‐L1 oligomerization. Plant Physiology, 187, 681–686. 34608964 10.1093/plphys/kiab305 PMC8491023

[mpp13195-bib-0057] Xia, B. , Fang, S. , Chen, X. , Hu, H. , Chen, P. , Wang, H. et al. (2016) MLKL forms cation channels. Cell Research, 26, 517–528. 27033670 10.1038/cr.2016.26 PMC4856759

[mpp13195-bib-0062] Yu, D.L. , Song, W. , Tan, E.Y.J. , Liu, L. , Cao, Y. , Jirschitzka, J. , et al. (2021) TIR domains of plant immune receptors are 2',3' ‐cAMP/cGMP synthetases mediating cell death. bioRxiv. 10.1101/2021.11.09.467869 35597242

[mpp13195-bib-0058] Yuan, M. , Jiang, Z. , Bi, G. , Nomura, K. , Liu, M. , Wang, Y. et al. (2021) Pattern‐recognition receptors are required for NLR‐mediated plant immunity. Nature, 592, 105–109. 33692546 10.1038/s41586-021-03316-6 PMC8016741

[mpp13195-bib-0059] Zhang, X. , Bernoux, M. , Bentham, A.R. , Newman, T.E. , Ve, T. , Casey, L.W. et al. (2017a) Multiple functional self‐association interfaces in plant TIR domains. Proceedings of the National Academy of Sciences of the United States of America, 114, E2046–E2052. 28159890 10.1073/pnas.1621248114 PMC5347627

[mpp13195-bib-0060] Zhang, Z.‐M. , Ma, K.‐W. , Gao, L. , Hu, Z. , Schwizer, S. , Ma, W. et al. (2017b) Mechanism of host substrate acetylation by a YopJ family effector. Nature Plants, 3, 17115. 28737762 10.1038/nplants.2017.115 PMC5546152

[mpp13195-bib-0061] Zhong, Y. & Cheng, Z.M. (2016) A unique RPW8‐encoding class of genes that originated in early land plants and evolved through domain fission, fusion, and duplication. Scientific Reports, 6, 32923. 27678195 10.1038/srep32923 PMC5039405

