# Supplementary figures and images for: Molecular insights into the biochemical functions and signalling mechanisms of plant NLRs
Source: Mol Plant Pathol. 2022 Mar 30;23(6):772–80. doi: 10.1111/mpp.13195 (PMC9104254; doi:10.1111/mpp.13195)

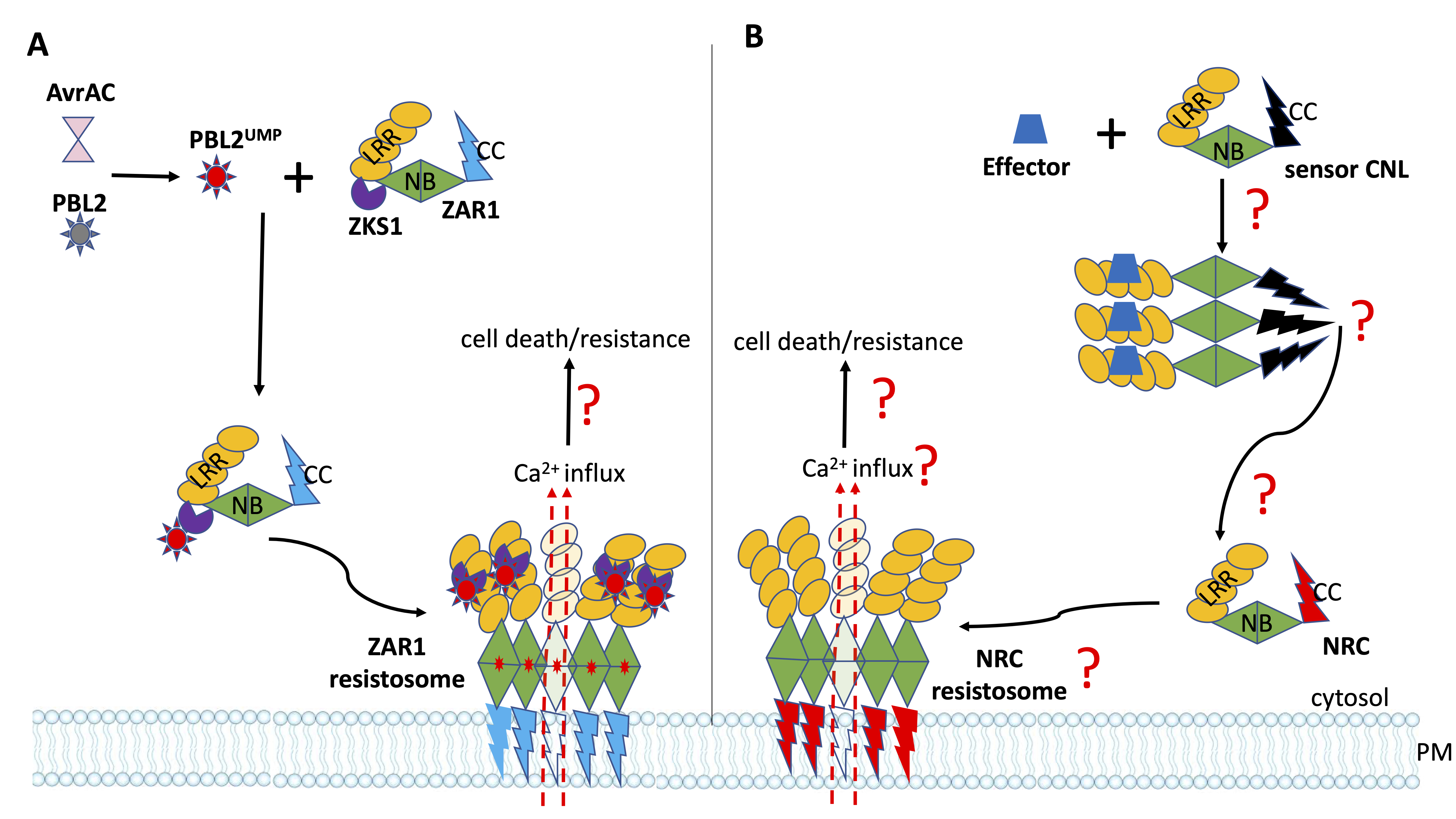

Supplement: Supplementary file 1 — Figure S1 CNL activation and signalling pathways. (a) AtZAR1 functions as an NLR singleton that does both jobs of sensor NLR and helper NLR to confer immunity and cause cell death. (b) Sensor CNLs on effector recognition activate immunity and cell death through NRC helpers that potentially function as Ca2+ channels [file MPP-23-772-s002.jpg]

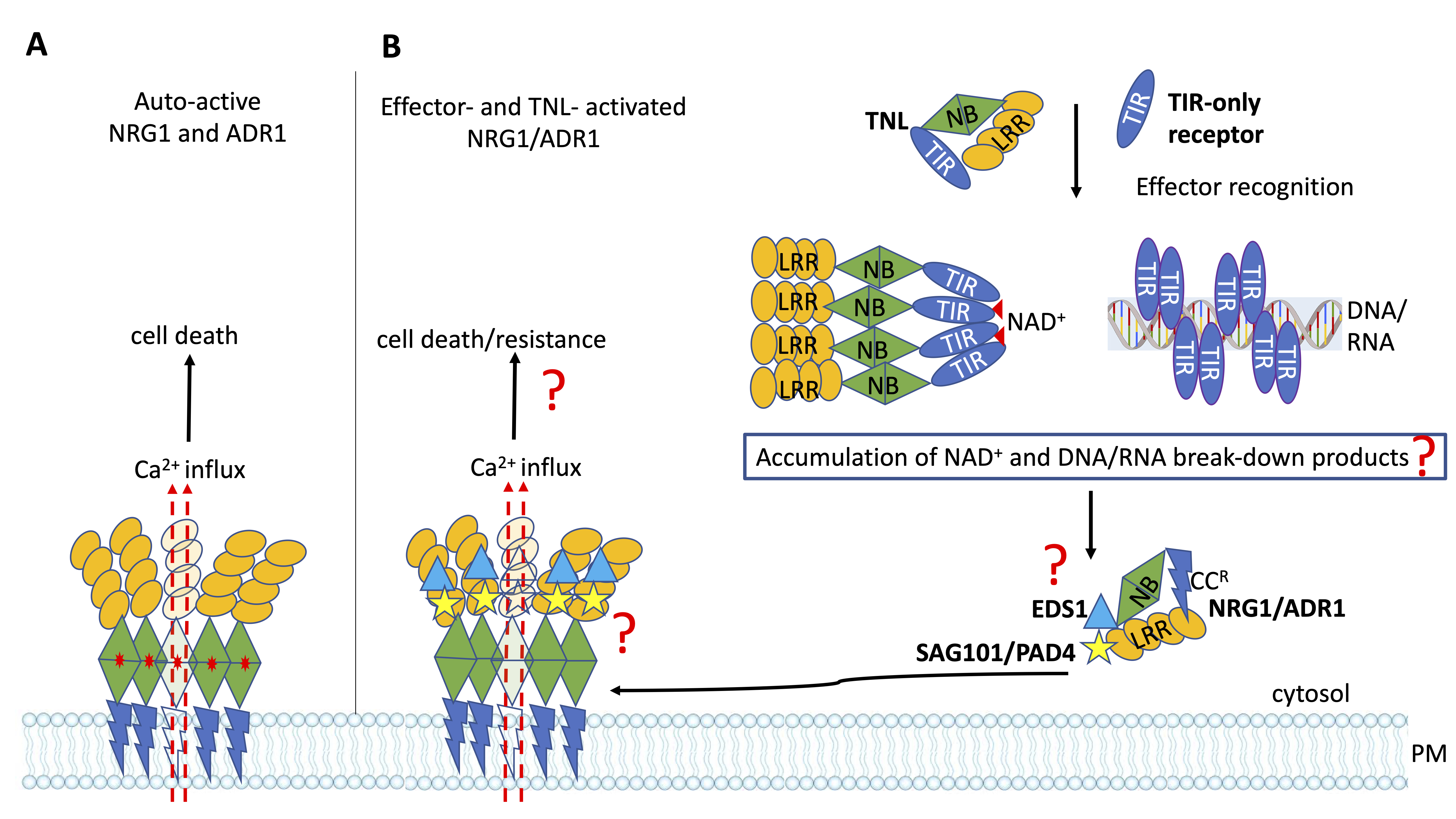

Supplement: Supplementary file 2 — Figure S2 TNL activation and signalling pathways. (a) Autoactive NRG1 and ADR1 function as Ca2+ channels to induce cell death. (b) TNL and TIR‐only receptors function as enzymes to produce small signalling molecules that activate complexes of EDS1/SAG101/NRG1 and EDS1/PAD4/ADR1, and potentially lead to NRG1 and ADR1 Ca2+ channel formation [file MPP-23-772-s001.jpg]
